# High glucose promotes gastric cancer chemoresistance *in vivo* and *in vitro*

**DOI:** 10.3892/mmr.2015.3522

**Published:** 2015-03-20

**Authors:** WEI ZHAO, RUI CHEN, MEI ZHAO, LIANG LI, LIN FAN, XIANG-MING CHE

**Affiliations:** 1 Department of General Surgery, The First Affiliated Hospital, College of Medicine, Xi’an Jiaotong University, Xi’an, Shaanxi 710061, P.R. China; 2 Department of Neonatal Surgery, The Children’s Hospital, Zhejiang University School of Medicine, Hangzhou, Zhejiang 310003, P.R. China; 3 Department of Pharmacology, College of Medicine, Xi’an Jiaotong University, Xi’an, Shaanxi 710061, P.R. China

**Keywords:** gastric cancer, diabetes, high glucose, chemoresistance, nicotinamide phosphoribosyltransferase

## Abstract

The aim of the present study was to determine whether gastric cancer chemoresistance was increased under high glucose conditions by means of a clinical case study and experimental cytology. The expression of nicotinamide phosphoribosyltransferase (Nampt), silent information regulator 1 (sirt1), p53, p-glycoprotein (P-gp) and topoisomerase (topo)-IIα was evaluated in gastric cancer tissues and gastric cancer with diabetes tissues by immunohistochemistry. Subsequently, the survival time of the patients was assessed. For further investigation, the human gastric cancer cell line SGC7901 was subjected to different glucose concentrations and the aforementioned proteins were detected using reverse transcription-quantitative polymerase chain reaction and western blot analysis. Finally, cell sensitivity to chemotherapy treatment was examined in order to elucidate the role of high glucose in MDR. Positive expression of Nampt, Sirt1, p53, P-gp and Topo-IIα was observed to be higher in gastric cancer with diabetes patients compared with gastric cancer patients (P=0.01, 0.003, 0.0025, 0.016 and 0.336, respectively) with reduced survival time. Similar results were observed in SGC7901 cells. Additionally, cell proliferation rates of SGC7901 cells increased at glucose concentrations of 4,500 and 9,000 mg/l. Notably, the inhibition rates of 5-fluorouracil on cells decreased over 48 h when treated with 4,500 and 9,000 mg/l glucose compared with 1,000 mg/l. In conclusion, patients suffering from gastric cancer and diabetes exhibited greater negative effects, such as a poorer response to chemotherapy and had a lower survival time. High glucose conditions promoted gastric cancer cell proliferation and reduced susceptibility to chemotherapy drugs. These data provided a potential diagnostic and therapeutic strategy for gastric cancer chemoresistance.

## Introduction

Gastric cancer is one of the most common types of malignancy, accounting for 8 and 10%, respectively, of malignant tumor total morbidity and mortality (1). Postoperative chemotherapy has been widely utilized following gastric cancer resection (2). However, multidrug resistance (MDR) is often the main cause for the failure of cancer treatment, particularly in gastric cancer (3). In addition, type 2 diabetes has been implicated in carcinogenesis and cancer progression (4). Despite studies investigating the mechanisms underlying the association between type 2 diabetes and cancer development (5,6), there is little information regarding the association between diabetes and MDR (7).

Nicotinamide phosphoribosyltransferase (Nampt) is essential for cell metabolism and survival (8). Bi *et al* (9,10) demonstrated that the level of Nampt in gastric cancer was higher than in normal tissue and that inhibition of Nampt may enhance the effects of chemotherapy. Nampt overexpression predicted a poor response to doxorubicin-based chemotherapy in patients with breast cancer (11,12). Silent information regulator 1 (Sirt1) is also known to be involved in cell proliferation, differentiation and apoptotic processes. Sirt1 overexpression is associated with a poor prognosis in gastric cancer (13,14). Nampt acts upstream of Sirt1, through the regulation of nicotinamide adenine dinucleotide (NAD) oxidation (15). However, the expression of Nampt and Sirt1 in gastric cancer with diabetes remains to be elucidated.

The aim of the present study was, therefore, to investigate MDR alterations in gastric cancer combined with type 2 diabetes. Subsequently, an *in vitro* model was established to investigate the role of high glucose in MDR.

## Materials and methods

### Chemicals and reagents

D-Glucose and methylthiazoly-diphenyl-tetrazolium bromide (MTT) were purchased from Sigma-Aldrich (St. Louis, MO, USA). The chemotherapy drug 5-fluorouracil (5-FU) was acquired from The First Affiliated Hospital, College of Medicine, Xi’an Jiaotong University (Xi’an, China). The primary antibodies used were as follows: Rabbit anti-human polyclonal anti-Nampt (AV42255; Sigma-Aldrich), rabbit anti-human polyclonal anti-p53 (sc-126; Santa Cruz Biotechnology, Inc., Santa Cruz, CA, USA)/p-glycoprotein (P-gp; sc-55510; Santa Cruz Biotechnology, Inc.), rabbit anti-human polyclonal anti-topoisomerase (topo)-IIα (20233-1-AP; Proteintech, Chicago, IL, USA) and rabbit anti-human monoclonal anti-Sirt1 (sc-15404; Santa Cruz Biotechnology, Inc.). The secondary antibodies and 3,3′-diaminobenzidine were purchased from ZSGB-BIO (Beijing, China).

### Patients and samples

A total of 68 patients with gastric cancer and 40 patients with gastric cancer with diabetes, who had undergone radical gastrectomy at The Department of General Surgery, The First Affiliated Hospital, College of Medicine, Xi’an Jiaotong University, between January 2000 and December 2004 were included in the present study. None of the patients had received preoperative neoadjuvant chemotherapy or radiotherapy. According to the tumor node metastasis classification of the International Union Against Cancer, those patients who were in stage I, II or III were matched based on gender and age. The control group was normal paired tissue samples collected from the patients from an area of tissue >5 cm away from the tumor. All studies were approved by the Ethics Committee of Xi’an Jiaotong University and every patient provided written informed consent prior to surgery. The baseline data of the study group are shown in Table I.

### Cell culture

The human gastric cancer cell line SGC-7901 was provided by the Fourth Military Medical University (Xi’an, China). Cells were cultured in low-glucose Dulbecco’s modified Eagle’s medium (1,000 mg/l glucose; Invitrogen Life Technologies, Carlsbad, CA, USA) supplemented with 10% fetal bovine serum (Hangzhou Sijiqing Biological Engineering Materials Co., Ltd., Hangzhou, China) and 100 IU/ml penicillin and 100 *μ*g/ml streptomycin (Harbin Pharmaceutical Group Co., Ltd, Harbin, China) at 37°C in a humidified atmosphere containing 5% CO_2_. Cells were subcultured with 0.25% Trypsin-EDTA (Amresco, LLC, Solon, OH, USA) once they reached >80% confluence.

### In vitro drug sensitivity assay

For the effective detection of chemotherapy drugs, cells cultured in 1,000, 4,500 and 9,000 mg/l glucose were subjected to 5-FU at 3.75, 15, 30, 60 and 120 *μ*g/ml. According to the results of daily cell counting, the growth curve with MTT was produced and the half maximal inhibitory concentration of each group was calculated.

### Immunohistochemical staining and scoring

The tissue samples were prepared cutting the paraffin-embedded tissue into serial sections 4 *μ*m thick and evenly cut into three sections (one for hematoxylin staining and the other two for immunohistochemical staining). The tissue sections were preserved in 2.5% glutaraldehyde-polyoxymethylene solution, were dehydrated and embedded in paraffin following routine methods and were immersed in distilled water. Subsequently, the paraffin sections were washed (3 × 5 min) in phosphate-buffered saline with Tween-20 (PBS-T; 0.01 M; pH 7.4) and then blocked with 0.3% H_2_O_2_-methanol at room temperature for endogenous peroxidase ablation. The following steps were conducted in a moist chamber. The non-specific binding sites were blocked by 30-min incubation in 5% normal goat serum (cat. no. ZLI-9022; Beijing Zhongshan Golden Bridge Company, Beijing, China) at room temperature for 20 min. The primary antibody was then added dropwise, then the sections were incubated for 2 h or overnight at 4°C followed by washing again with in PBS-T (3 × 5 min). According to the PV-6000 direction to add the reagent, the sections were incubated for 30 min at room temperature, followed by washing with PBS-T(3 × 5min). The sections were then stained with 3,3-diaminobenzidine (cat. no. ZLI-9017; Beijing Zhongshan Golden Bridge Company), were kept at room temperature without light for 10 min and were immersed in distilled water for 10 min. The sections were then stained with hematoxylin for 2 min, completing the staining with the distilled water for 10 min. Dehydration of the sections was then conducted with sequential 1-min ethanol washes starting with 75%, followed by 80% and finishing with an 100% ethanol wash. The sections were mounted with neutral gum and then sealed and analyzed by optical microscopy (Nikon 80i; Nikon Corporation, Tokyo, Japan). The intensity of immunostaining was assessed by two different observers, with respect to the staining intensity into the following four grades: −, no staining (negative); +, faintly stained up to 25% (weak positive); ++, 25-50% cells moderately stained (moderate positive); +++, 50% or more cells markedly stained (strong positive) (16).

### RNA extraction and reverse transcription quantitative polymerase chain reaction (RT-qPCR)

Total RNA was isolated using the RNA Fast 200 purification kit from Fastagen Biotech (Shanghai, China) according to the manufacturer’s instructions. Reverse transcription of RNA was performed with ExScriptTM RT reagent kit (Takara Biotechnology Co., Ltd., Dalian, China). The cDNA was then subjected to RT-PCR. All primer pairs shown in Table II were designed by Oligo 6.0 primer analysis software (Oswel Research Products, Beijing, China). qPCR was performed in duplicate reactions of 12.5 *μ*l volume SYBR II (Takara Biotechnology Co., Ltd.), 1 *μ*l each of specific forward and reverse primers and 9.5 *μ*l RNase Free H_2_O up to 24 *μ*l. Samples were heated for 85°C followed by 40 cycles of amplification for 15 sec at 95°C and 1 min at 60°C. The mRNA level of β-actin was measured as an internal reference.

### Protein extraction and western blot analysis

Cells were treated with lysis buffer. Protein samples (30 *μ*g) were separated by 10% gels and then transferred onto a polyvinylidene difluoride membrane (Sterlitech Corp., Kent, WA, USA). Blots were probed with antibodies against Nampt (1:1,000), Sirt1 (1:500), p53 (1:500), P-gp (1:200) and Top-IIα (1:200). Following primary antibody incubation, membranes were incubated with horseradish peroxidase-conjugated secondary immunoglobulin G (1:2,000). The immunoreaction was visualized using ECL-Plus reagent (Pierce Biotechnology, Inc., Rockford, IL, USA) and analyzed using a Gel-Pro Analyzer (Media Cybernetics, Silver Spring, MD, USA).

### Statistical analysis

The data are presented as the mean ± standard error of the mean. Statistical analysis was performed using SPSS 16.0 software (SPSS, Inc., Chicago, IL, USA). Data were analyzed using a χ^2^ test. P<0.05 was considered to indicate a statistically significant difference. Correlations were analyzed using Spearman’s rank correlation coefficient test.

## Results

### Alterations in the levels of chemoresistance protein markers in gastric cancer with diabetes

**Figure 1:**
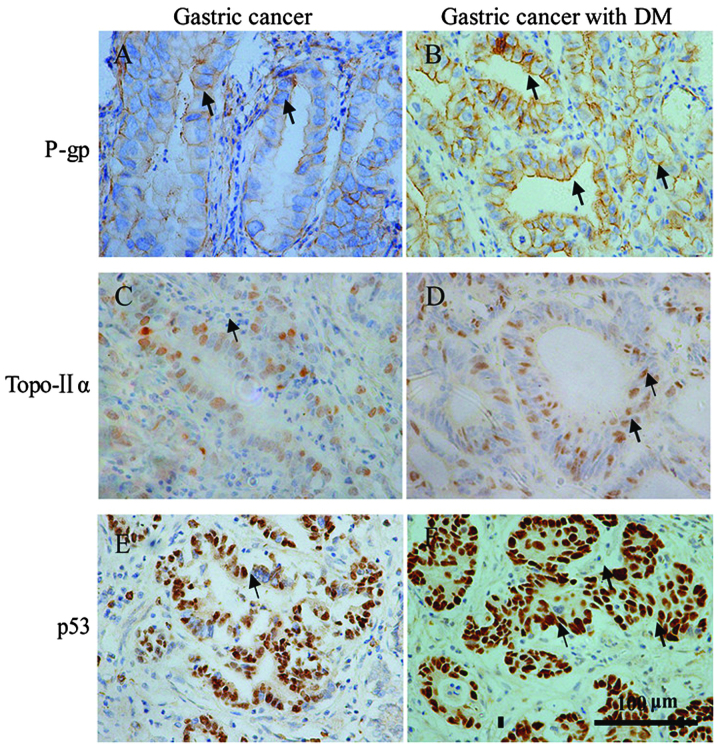
Representative images of immunohistochemical staining with diaminobenzidine and hematoxylin in gastric cancer and gastric cancer with DM. (A and B) P-gp was positive in the cytomembrane. (C and D) Top-IIα and (E and F) p53 exhibited nuclear positivity. P-gp, p-glycoprotein; topo-IIα, topoisomerase-IIα; DM, diabetes mellitus.

At present, P-gp and p53 are recognized as typical chemoresistance protein markers (17,18). The present study demonstrated increased levels of P-gp and p53 in patients with gastric cancer with diabetes compared with gastric cancer alone (Fig. 1 and Table III). It is well established that Topo-IIα is a target for anti-cancer drugs (19). Notably, the level of Topo-IIα in gastric cancer with diabetes tissues was lower than that in gastric cancer alone (Fig. 1 and Table III). p53 and Topo-IIα were found primarily in the nuclei, while P-gp was identified in the cytomembrane, which is consistent with previous studies (20,21). The clinical pathological features are summarized in Table III.

### Diabetes increases the expression of Nampt and Sirt1 in gastric cancer and decreases survival rate

**Figure 2:**
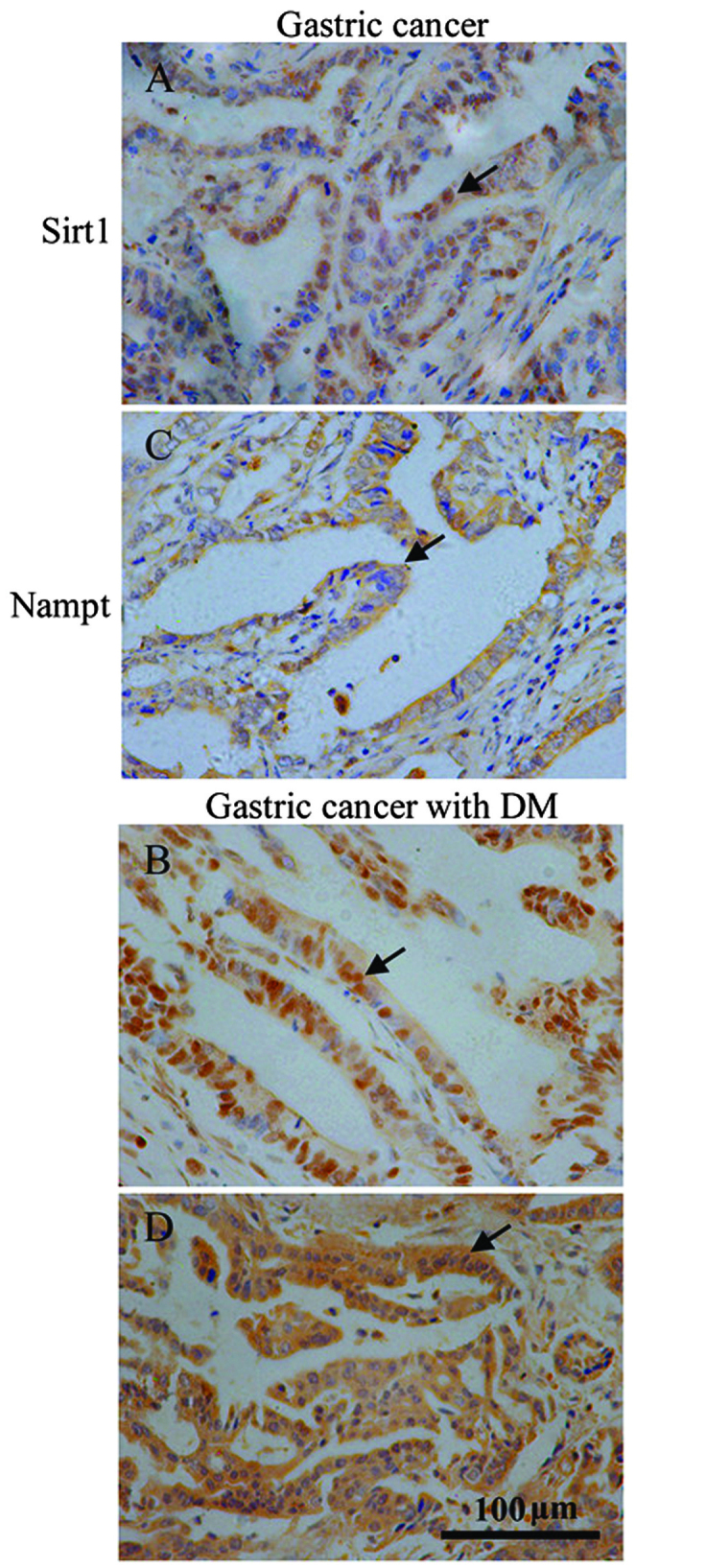
Representative images of immunohistochemical staining with diaminobenzidine and hematoxylin in gastric cancer and gastric cancer with DM. (A and B) Sirt1 exhibited nuclear positivity. (C and D) Nampt exhibited positive staining in the cytoplasm. Sirt1, silent information regulator 1; Nampt, nicotinamide phosphoribosyltransferase; DM, diabetes mellitus.

**Figure 3:**
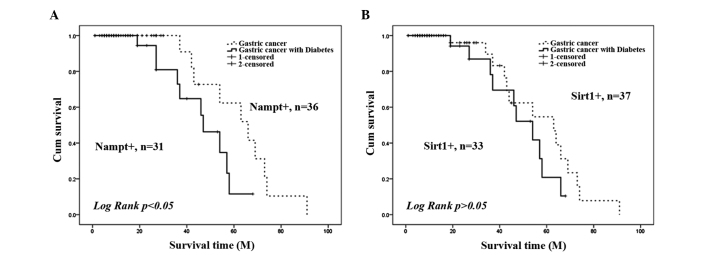
Survival analysis in gastric cancer and gastric cancer with DM. Effects of (A) Nampt and (B) Sirt1 on overall survival rate in gastric cancer and gastric cancer with DM groups. Sirt1, silent information regulator 1; Nampt, nicotinamide phosphoribosyltransferase; DM, diabetes mellitus.

To further investigate the mechanism underlying chemoresistance, the present study examined whether Nampt and Sirt1 were involved in the process. Levels of Nampt and Sirt1 were observed to be higher in gastric cancer with diabetes than gastric cancer tissues (Fig. 2 and Table III). To further investigate the impact on survival time, the survival curve was also examined. Increased Nampt was associated with reduced overall survival rate of gastric cancer with diabetes by univariate analysis (Fig. 3). However, Sirt1 had little effect on the overall survival rate (Fig. 3).

### High glucose induces the expression of Nampt, Sirt1, p53, P-gp and inhibits Top-IIα in the gastric cancer cell line SGC7901

**Figure 4:**
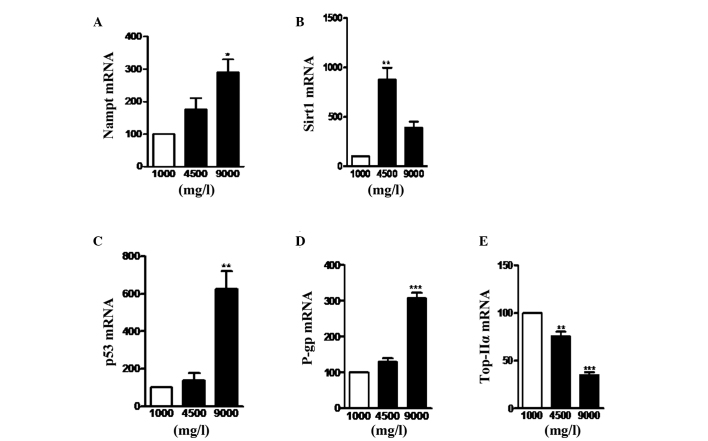
Effect of high glucose on the mRNA expression of Nampt, Sirt1, p53, P-gp and Top-IIα. SGC7901 cells were plated in 6-well plates and then incubated for 72 h in the presence of high glucose (4,500 and 9,000 mg/l) in order to examine Nampt, Sirt1, p53, P-gp and Top-IIα gene expression using reverse transcription quantitative polymerase chain reaction. Treatment of SGC7901 cells with high glucose resulted in increased (A) Nampt, (B) Sirt1, (C) p53, (D) P-gp and (E) decreased Top-IIα mRNA levels. Data are presented as the mean ± standard error of the mean (n=6). ^*^P<0.05, ^**^P<0.01 and ^***^P<0.001, v.s. the 1,000 mg/l group. Sirt1, silent information regulator 1; Nampt, nicotinamide phosphoribosyltransferase; P-gp, p-glycoprotein; topo-IIα, topoisomerase-IIα.

**Figure 5:**
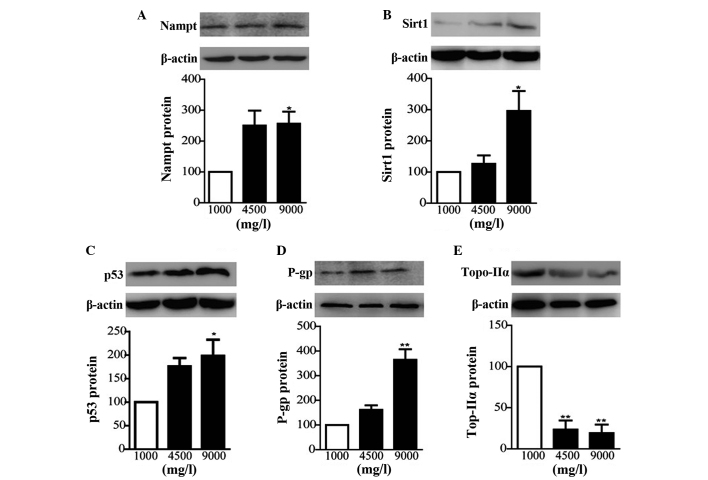
Effect of high glucose on the protein expression of Nampt, Sirt1, p53, P-gp and Top-IIα. Gastric cancer SGC7901 cells were plated in 6-well plates and incubated for 72 h in the presence of high glucose (4,500 and 9,000 mg/ml) to examine Nampt, Sirt1, p53, P-gp and Top-IIα protein expression patterns. Total protein was extracted from cells and analyzed by western blot analysis. The protein expression of (A) Nampt, (B) Sirt1, (C) p53 and (D) P-gp was significantly increased following treatment with 9,000 mg/ml glucose in SGC7901 cells, however, the expression of (E) Top-IIα was significantly decreased. The data are expressed as the mean ± standard error of the mean (n=6). ^*^P<0.05 and ^**^P<0.01, high glucose v.s. the 1,000 mg/l glucose group. Sirt1, silent information regulator 1; Nampt, nicotinamide phosphoribosyltransferase; P-gp, p-glycoprotein; topo-IIα, topoisomerase-IIα.

PCR analysis indicated that cells treated with glucose at 4,500 and 9,000 mg/l exhibited increased mRNA expression of Nampt, Sirt1, p53 and P-gp compared with 1,000 mg/l. In addition, the levels of Topo-IIα were reduced at 4,500 and 9,000 mg/l glucose (Fig. 4). Western blot analysis confirmed these effects at the protein level (Fig. 5). These results suggested that high glucose induced chemoresistance protein markers at the transcriptional and translational levels.

### High glucose results in chemoresistance of gastric cancer cells treated with 5-FU

**Figure 6:**
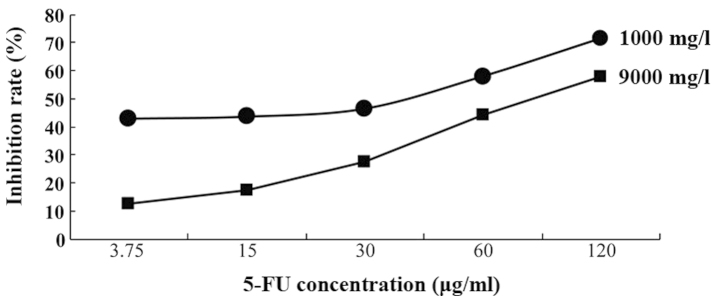
Combination of 5-FU with high glucose increased inhibition of gastric cancer cells. 5-FU, 5-fluorouracil.

Subsequently, it was examined whether high glucose levels induced chemoresistance to 5-FU in SGC-7901 cells. The number of live cells was measured using an MTT assay. When exposed to 5-FU, SGC7901 cells exhibited a higher cell proliferation rate in the presence of high glucose (9,000 mg/l) compared with lower glucose concentrations (1,000 mg/l) over 48 h, suggesting that high glucose attenuated the growth inhibitory effect of 5-FU (Fig. 6).

## Discussion

Cancer with type 2 diabetes appears to result in a higher risk of MDR (22). The present study predominantly focused on the effects of high glucose on gastric cancer. Initially, it was identified that chemoresistance protein markers, including P-gp and Topo-IIα in diabetes differed from that of cancer alone. Secondly, high glucose increased Nampt and Sirt1, which may provide a possible mechanism for MDR in patients and a lower inhibition rate with anticancer drugs in the cancer cell line. The present findings are in line with previous studies concerning the association between diabetes and the prevalence of cancer (23,24).

The wider effects of type 2 diabetes, including hyperglycemia and hyperinsulinemia can lead to cancer progression (25). Based on these studies, a mechanism was postulated to explain the possible promoting impact of diabetes on gastric cancer; hyperglycemia may promote the expression of Nampt/Sirt1 in gastric cancer tissue, thereby upregulating the expression level of mutated p53 in tumor cells. Therefore the possible mechanisms by which MDR occurs may proceed through promoting P-gp expression and reducing Topo-IIα expression.

Several studies have observed overexpression of Nampt in gastric cancer and Nampt inhibition has been found to suppress cancer cell growth and induce apoptosis (26). Nampt level in the plasma was higher in the diabetic population compared with healthy controls (27). In line with these studies, in the present study the expression of Nampt in gastric cancer patients with diabetes was found to be higher than that identified in gastric cancer alone. It is well established that Nampt dictates NAD^+^ in mammalian cells and NAD^+^ is central to cell proliferation (28). Nampt promoted cancer cell proliferation through elevated NAD^+^ synthesis. Therefore, Nampt overexpression represents a key mechanism in tumor biology.

Sirt1 is a conserved stress response protein involved in a broad range of biological processes (29). Previous studies have demonstrated that Sirt1 is involved in the development of MDR (30,31). Furthermore, Nampt has been demonstrated to enhance Sirt1 activity by increasing NAD^+^ (6). C-MYC also contributed to Sirt1 activation in breast cancer (32). Other studies have also demonstrated that Sirt1 is important in promoting cell growth and chemoresistance in PC3 and DU145 cells (33,34). Similarly, in the present study it was revealed that Sirt1 expression was upregulated under high glucose conditions. These data indicated that Sirt1 is possibly an import mediator of MDR caused by high glucose and a valuable therapeutic target for gastric cancer.

In order to verify the possible underlying mechanism using cytology, the human gastric cancer cell line SGC7901 was subjected to high glucose medium. Consistent with *in vivo* results, the hyperglycemic condition promoted the expression of Nampt and Sirt1, then increased the expression of mutated p53 resulting in an upregulation of P-gp and a downregulation of Topo-IIα. In addition, the high glucose condition promoted gastric cancer cell proliferation and reduced susceptibility to chemotherapy drugs.

Nampt may elevate Sirt1 expression and activity through the glucose metabolic pathway. p53, as a tumor suppressor, is able to induce tumor cell apoptosis and arrest the cell cycle. Sirt1 may inactivate the 382nd lysine residue of p53, reduce the expression of the wild-type p53 protein combining with the DNA cis element to promote p53 mutation. Compared with that of wild-type p53, the mutated p53 has a longer half-life, which is conductive to observation and evaluation. Overexpression of mutated p53 in tumor cells is positively correlated with higher levels of expression of P-gp, an ATP-dependent protein, resulting in MDR (35).

In conclusion, it was demonstrated that type 2 diabetes conferred MDR to gastric cancer and this effect was at least partially mediated by increased Nampt and Sirt1 expression levels. Thus, in the future, postoperative chemotherapy needs to utilize a combination therapy of gastric cancer treatment and diabetes treatment. In addition, these findings provide novel insights into the role of Nampt and Sirt1 in MDR.

## Figures and Tables

**Table I:** Characteristics of patients.

Characteristic	Gastric cancer	Gastric cancer with DM
Gender		
Male	50	30
Female	18	10
Age (years)	59.2±18.4	64.1±14.6
Histology		
Differentiated	56	34
Poorly differentiated	12	6
TNM staging		
I+II	35	11
III+IV	33	29

DM, diabetes mellitus; TNM, tumor node metastasis.

**Table II:** Gene sequences for quantitative polymerase chain reaction.

Human gene	Forward primer	Reverse primer
Nampt	5′-AAGAGACTGCTGGCATAGGA-3′	5′-ACCACAGATACAGGCACTGA-3′
Sirt1	5′-TTGTACGACGAAGACGAC-3′	5′-TAGAGCTTGCATGTGAGG-3′
p53	5′-TCAGTCTTCCTCCAACC-3′	5′-GGAAAAGACTGAAGGGTG-3′
P-gp	5′-CATTCCTCCTGGAAATTCAACCT-3′	5′-CTTCAAGATCCATTCCGACCTC-3′
Topo-IIα	5′-TGGAAACAGCCAGTAGAG-3′	5′-ATCTTTGTCCAGGCTTTG-3′
β-actin	5′-CCTGGGCATGGAGTCCTGTG-3′	5′-TCTTCATTGTGCTGGGTGCC-3′

Sirt1, silent information regulator 1; Nampt, nicotinamide phosphoribosyltransferase; GC, gastric cancer; P-gp, p-glycoprotein; topo-IIα, topoisomerase-IIα.

**Table III:** Expression of Nampt, Sirt1, p53, P-gp and Top-IIα between two groups using χ^2^ test.

Protein	Positive cases	Negative cases
Nampt		
GC	36	32
GC with diabetes	31	9
Sirt1		
GC	37	31
GC with diabetes	33	7
p53		
GC	33	35
GC with diabetes	29	11
P-gp		
GC	35	33
GC with diabetes	30	10
Topo-IIα		
GC	23	45
GC with diabetes	12	28

Sirt1, silent information regulator 1; Nampt, nicotinamide phosphori-bosyltransferase; GC, gastric cancer; P-gp, p-glycoprotein; topo-IIα, topoisomerase-IIα.
